# Climbing performance in males: the importance of climbing-specific finger strength

**DOI:** 10.1007/s00421-025-05802-5

**Published:** 2025-05-05

**Authors:** B. F. Buraas, M. F. Brobakken, E. Wang

**Affiliations:** Department of Health and Social Sciences, Molde University College, Britvegen 2, 6410 Molde, Norway

**Keywords:** Bouldering, Rock climbing, Forearm, Hangboard, Campusboard, Redpoint

## Abstract

**Purpose:**

Climbing is one of the fastest growing sports worldwide and with recent inclusion in the Olympic Games, and mounting number of indoor climbing gyms, its popularity is augmented. However, which physiological factors that predominantly determine climbing performance is unclear. Surprisingly, evidence of the importance of climbing-specific finger strength, intuitively the most obvious physiological component for climbing performance, is limited. This study sought to examine the relationship between distal finger digits isometric strength, assessed hanging from a 22 mm edge, and redpoint climbing and bouldering performance. Moreover, the aim was to contrast the results to less climbing-specific forearm flexor (handgrip) strength and multi-joint upper extremities (pullup) strength.

**Methods:**

Nineteen males (26 ± 3 years; 181 ± 7 cm; 75.2 ± 7.7 kg) with a previous redpoint level from 6b + to 8c (French grading scale) participated in the study.

**Results:**

Very strong and strong associations, respectively, were shown between climbing-specific finger strength and bouldering (*r* = 0.89) and redpoint (*r* = 0.67, both *p* < 0.01) climbing performance. It also exhibited a strong correlation with campus boarding (*r* = 0.82, *p* < 0.01). Handgrip strength was moderately associated with redpoint (*r* = 0.54) and campus board (*r* = 0.47, both *p* < 0.05), but not bouldering performance (*p* > 0.05). Pullup-strength exhibited a moderate association with bouldering (*r* = 0.55) and campus boarding (*r* = 0.57, both *p* < 0.05), but not redpoint performance. Body weight associated moderately with bouldering (*r* = − 0.49) and campus boarding (*r* = − 0.51, both *p* < 0.05), whereas height did not (all *p* > 0.05).

**Conclusion:**

Isometric distal finger strength appears to be the physiological factor most strongly related to bouldering and redpoint performance in males. However, it should be assessed in a climbing-specific exercise.

## Introduction

The International Federation of Sport Climbing (IFSC) reported in 2019 that there were 44.5 million climbers worldwide (IFSC 2019). With inclusion into the Olympic Games in 2021 and 2024, the popularity was substantially augmented, with number of climbers and media coverage rocketing. The global climbing gym market size is estimated to reach $1.78 billion by 2026 and only in the USA ~ 60 new climbing gyms open annually (Worldmetrics 2024). Yet, evidence of which physiological factors that have a critical impact on climbing performance, and how they may be trained, is limited. Perhaps, at least in part, owing to the fact that climbing demands a symphony of muscle strength, endurance, and flexibility, with the relative contribution of these distinctly different physiological components likely evolving with the recent development of the sport. Advancing the climbing knowledge of exercise physiology likely has a bearing on our understanding of the sport.

Anthropometric characteristics, muscle strength, endurance, flexibility, and body composition have all been shown to influence climbing performance (Giles et al. 2006). However, it appears, to date, the relative contribution of these physiological factors is elusive, and if any single factor may be of key importance, or not just simply be different from what is observed in non-climbers. Notably, most previous studies also rely on self-reported previous climbing performance, which may cloud the strength of associations (Baláš et al. 2012; Giles et al. 2021).

Forearm flexor skeletal muscle strength, typically referred to as “finger strength”, is intuitively a physiological factor that should be of key importance for performance, since climbers, whether bouldering or lead climbing, spend much of their time hanging from their fingers during steep climbing. A firm grip enables the climber to propel towards the next holds, and remarkable individual finger strength performances have been reported in the best climbers in the world, such as being able to hang from the distal digits of one hand with an additional excess of > 30 kg in addition to body weight. In line with this notion, several studies have documented a difference in finger strength (Baláš et al. 2014; Fryer et al. 2015a, b; Giles et al. 2021) and handgrip strength (Macdonald and Callender 2011) between climbers and non-climbers or between climbers with a self-reported low- and high-performance level. Although an association is likely in a heterogonous sample with extreme ends of the performance spectrum, i.e., trained vs. untrained, it remains uncertain if such a relationship exists, and with which strength, in climbers of various ability. However, evidence from studies aiming to investigate the role of finger strength in various climbing disciplines is scarce.

Given the potentially critical importance of finger strength for climbing performance, further detailed investigation of this physiological factor is warranted. Thus, the current study aimed to examine climbing-specific finger strength, assessed as hanging from a 22-mm, declining 30 degrees, edge with the distal finger joint and its association with current bouldering and redpoint performance, respectively, as well as previous self-reported climbing performance. As forearm flexor muscle composition is highly complex, the aim was also to contrast the climbing-specific finger strength to more general forearm flexor strength (assessed as handgrip) and multi-joint upper extremities strength (measured as pullup strength) to examine the potential necessity of testing muscle strength in a climbing-specific fashion. Specifically, we hypothesized that climbing-specific finger strength would exhibit a strong association with both bouldering and redpoint climbing performance in moderate to high level individuals, while handgrip and pullup strength would not.

## Methods

### Participants

Twenty males with a previous redpoint level from 6b + to 8c (French grading scale), the last 2 years, were enrolled in the study from a local climbing community in Trondheim, Norway. One subject withdrew from the study after inclusion due to a non-climbing related injury. The subject characteristics for those that completed the study are given in Table 1. The inclusion criteria were being healthy, non-smoking, and climbing for ≥ 2 years with a self-reported climbing ability of ≥ 6b + in redpoint or alternatively ≥ 6 A in bouldering. It was chosen to only include males, to minimize the influence of sex differences, and due to more male climbers available for enrollment at the time. The exclusion criteria were any cardiovascular, pulmonary or musculoskeletal disease or/and any known physical handicap or medication that would influence climbing performance. The voluntary recruited participants signed informed consents and were informed that they could withdraw any time without giving reason. A local institutional ethics board approved that the study met ethical standards, and assured it was carried out in accordance with the Helsinki Declaration.

**Table 1:** Subject characteristics

	Mean	Standard deviation	Range	Coefficient of variation (%)
Age, years	26	3	19–31	11.5
Body weight, kg	75.2	7.7	61–88	10.2
Height, cm	181	7	167–194	3.9
Body mass index, kg ∙ m^−2^	23.1	1.6	20.1–26.9	6.9
Self-reported best performance				
Climbing grade*	7b +	–	6b + – 8c	–
Climbing grade converted**	9.3	4.8	1–16	51.6

*n* = 19

*Highest redpoint rating of the season

**Rating converted to numeric scale (1 = 7-, 20 = 10-/10)

### Study outline

**Fig. 1:**
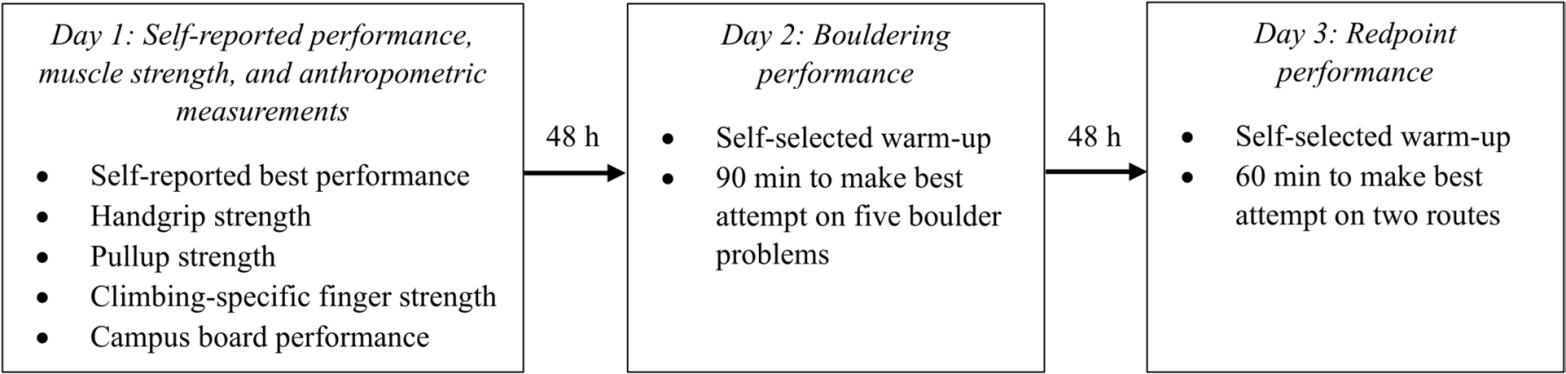
Schematic illustration of the test routines in the current study

Testing was conducted over three separate days consisting of muscle strength and anthropometric measurements, bouldering performance, and redpoint performance, respectively (Fig. 1). In an attempt to reduce the influence of non-physiological factors, such as tactical and technical aspects, climbing performance was not tested on slabs, and climbers were given time to practice the boulders and routes. The testing days were separated by 48 h of rest. The participants were also instructed to refrain from moderate to heavy physical activity at least 48 h prior to the testing, and during the recovery periods between testing days. The encouragement to perform maximally was given to participants during all physical testing. Values for all strength tests are given in absolute values and not percentages, as excess kg over the individuals’ body weight is most appropriate to determine climbing performance.

### Muscle strength testing procedures

#### Handgrip strength

Testing of muscle strength started with measurements of handgrip isometric strength on each of the arms individually, with an electronic device that registers grip force with strain gauge (Grippit, AB Detektor, Sweden). The device consists of an elliptical handle (12.5 cm in circumference) and an electronic unit able to document maximal force (0–1000 N; resolution of 1 N) in intervals of 0.5 s (Hammer and Lindmark 2003; Nilsen et al. 2012). The subjects sat on a chair beside a table, with their elbow in a ~ 90 degrees angle, shoulder in a neutral rotational position, and the arm placed in a fixed setup to minimized involvement from other muscle groups. The wrist was 0–30 degrees dorsiflexed and 0–15 degrees ulnar deviated. The subjects were familiarized with the handgrip device and performed two warm-up sets of light to moderate contractions. Subsequently, three measurements of each arm were performed, with one minute of rest in between. Their best result for right and left hand, respectively, was recorded, and their body mass subtracted from the results to comply with pullup and climbing-specific finger strength testing.

#### Pullup strength

The participants warmed up for ~ 15 min with general and specific low to moderate intensity exercises before the remaining strength tests. Pullups were performed without shoes and with the forearms pronated, and shoulder width distance between the hands. Following the warm-up procedure the load was gradually increased by adding weights (Eleiko, Sweden), with increments of 2.5–20 kg, attached to the individuals climbing harness with a sling and a carabiner, and one repetition maximum (1RM) reached within five attempts. Three minutes rest periods were given between each attempt. An approved attempt had to start with straight arms and end with the chin above the 22 mm bar, and no movement was allowed with legs or other parts of the body other than the targeted muscle groups.

#### Climbing-specific finger strength

With the same position as when starting the pullup, the participants were hanging from an edge, 22 mm deep at 30 degrees from horizontal, with both arms. Individuals could choose their grip type in accordance with personal preference. Similarly to pullup, the load was gradually increased by attaching 2.5–20 kg weights to the subjects’ harness. A minimum of 5 s hanging was required for an attempt to be approved.

### Campus board

The participants were instructed to hang with straight arms in shoulder width position, without any support for the legs, from campus rung (Metolius, USA) nr 1. The board had an overhanging inclination of 15 degrees from vertical, and the distance between the rungs were 22 cm. The subjects were then instructed to, with two arm movements in preferred order, to reach the highest rung possible on the campus board. The best out of three attempts was recorded.

### Bouldering performance

After individual self-selected warm up procedures, participants had 90 min to make their best effort to complete five boulder problems made by route setters blinded to the identity of the participants. The route setters were instructed to create problems including the most common grip positions, i.e., crimp, half crimp, open hand, sloper, pinch, and pocket. They were also encouraged to create problems requiring different skills and styles, such as compression, dyno, balance, and finger movements, at inclinations from vertical to 50 degrees overhang. Each problem became progressively more difficult, and the score increased for every hold the subjects were able to reach and control. An extra point was given if the participants were able to complete a boulder problem at first attempt (flash). The scores used for analyses were based on the participants’ best attempt and adjusted to represent a numeric scale from 0 to 100.

### Redpoint performance

After self-selected, individual, warm-up procedures, the subjects attempted two climbing routes, with progressive difficulty between routes. The routes were created on an indoor 12-m-high, 15 degrees overhanging, climbing wall (Bendcrete, England). Again, as for the bouldering problems, routes setters aimed to include a variety of grips, styles and movements. They also aimed to make the routes with continuous difficulty, i.e., without good resting positions. The subjects were given 1 h to try the routes and make their best attempt on each of the routes, a discipline typically referred to as redpoint climbing. However, to make the scenario as similar as possible for each climber, they were not allowed to watch other climbers try the routes. Every established hold higher on the route yielded a higher score. Time was measured during the climbing, starting when the individual left the ground. If climbers reached the same high-point, higher rank was given to the fastest climbers. For consistency, as with bouldering performance, the scores used for analyses were based on the participants’ best attempt and adjusted to represent a numeric scale from 0 to 100.

### Statistical analyses

Data are presented as mean and standard deviation, unless otherwise stated. All statistical analyses were done with SPSS Statistics v. 29.0 (IBM Corp.; Armonk, NY, USA) and figures constructed with GraphPad Prism 10 (GraphPad Software; Boston, MA, USA). The assumption of normality of data, assessed by the Shapiro–Wilk test, was not violated for any of the data included in correlation analyses. As such, the linear association between variables was examined with Pearson correlation coefficients, with results presented in either r or r^2^ values. Correlations were classified as moderate, strong, or very strong between 0.5–0.7, 0.7–0.9, and 0.9–1.0, respectively (Hinkle et al. 2003). The normality of residuals were assessed by visual inspection of Q-Q-plots for regression analyses. The sample size estimation was performed applying a correlation coefficient between climbing-specific finger strength and redpoint performance of 0.2 and 0.8 (Baláš et al. 2012), respectively, for the null and alternative hypotheses (80% power and alpha level of 0.5), yielding an estimated minimum of 14 subjects. Coefficient of variation (%) was also calculated for the outcomes. To indicate statistical significance, a two-tailed *p*-value level of < 0.05 was applied.

## Results

For all 19 included subjects, mean age was 26 ± 3 years, with body weight and height of 75.2 ± 7.7 kg and 181 ± 7 cm, respectively. The mean self-reported best redpoint grade of the season was 7b +, ranging from 6b + to 8c. Handgrip strength for the right and left arms was 66 ± 8 (652 ± 79 N) and 63 ± 9 kg (617 ± 84 N), respectively, yielding a total handgrip strength of 129 ± 16 kg (1269 ± 154 N).

**Fig. 2:**
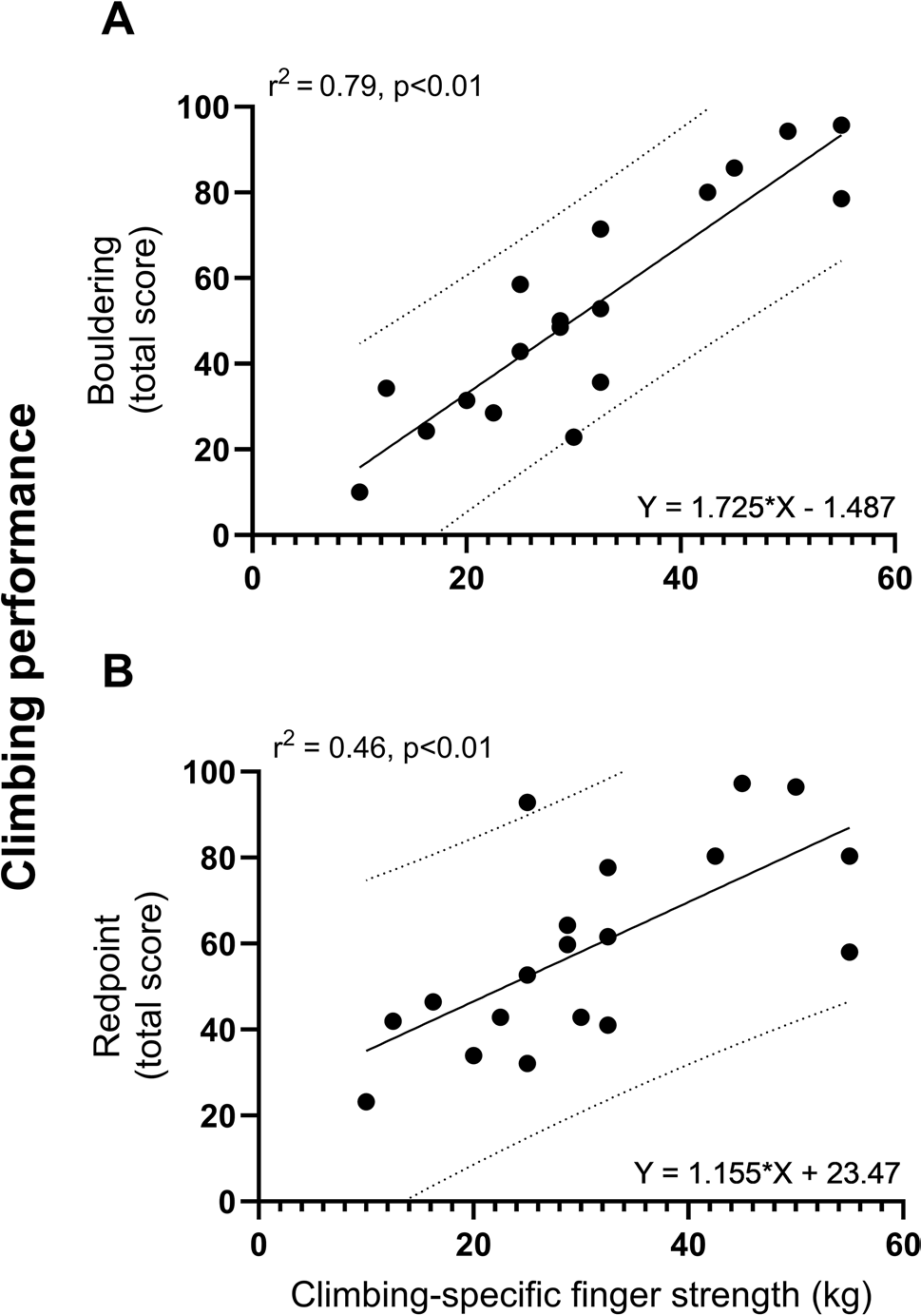
The relationship (Pearson correlation coefficient squared, *r*^2^, and regression equation) between climbing-specific finger strength (kg) and **A** bouldering (total score) and **B** redpoint (total score) climbing performance. *n* = 19

**Fig. 3:**
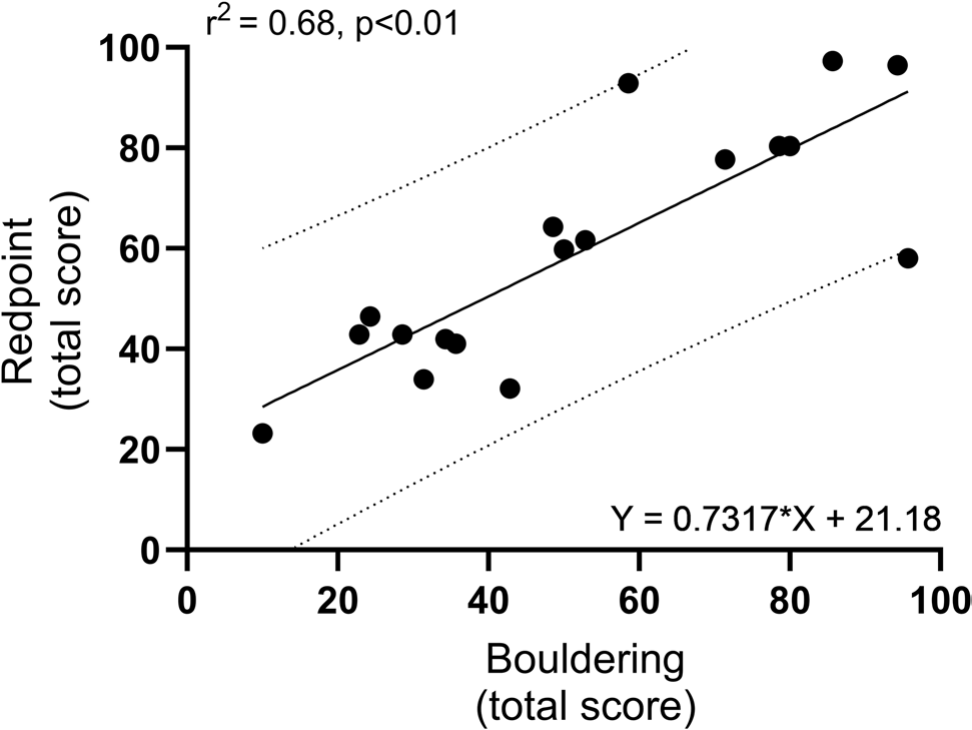
The relationship (Pearson correlation coefficient squared, r^2^, and regression equation) between bouldering (total score) and redpoint (total score) climbing performance. *n* = 19

Climbing-specific finger strength associated very strongly (*r*^2^ = 0.79) and strongly (*r*^2^ = 0.46) with bouldering and redpoint climbing performance, respectively (Table 2; Fig. 2 A and B). Climbing-specific finger strength also associated strongly (*r*^2^ = 0.67) with campus board score (Table 3). Forearm flexor handgrip strength was associated moderately with redpoint climbing (*r*^2^ = 0.29) and campus board (*r*^2^ = 0.22) scores, but not bouldering performance (*p* > 0.05). The pullup strength was associated moderately with bouldering (*r*^2^ = 0.30) and campus board (*r*^2^ = 0.33), but not redpoint performance (*p* > 0.05). This was mirrored by body weight which was also found to associate moderately (negatively) with bouldering (*r*^2^ = 0.24) and campus board (*r*^2^ = 0.26), but not redpoint performance (*p* > 0.05). Body mass index (BMI) was also associated moderately (negatively) with redpoint (*r*^2^ = 0.21) and campus board (*r*^2^ = 0.23) performance. Height, however, was not associated with climbing performance (all *p* > 0.05). Finally, the relationship between bouldering and redpoint performance showed a strong association (*r*^2^ = 0.68) (Fig. 3).

**Table 2:** Muscle strength and climbing performance

	Mean	Standard deviation	Range	Coefficient of variation (%)
Muscle strength*				
Finger strength, kg	31	13	10–55	41.9
Handgrip strength				
kg	54	17	26–81	30.5
N	532	162	255–794	30.5
Pullup strength, kg	38	9	25–60	23.7
Climbing performance				
Redpoint climbing**				
Route 1				
Score	41	11	20–50	26.8
Time, s	128	36	85–210	28.1
Route 2				
Score	19	15	4–47	79.0
Time, s	75	49	15–178	65.3
Total score both routes	60	23	23–97	38.3
Bouldering, total score§	53	26	10–96	49.1
Campus board, score§§	14	7	1–25	50.0

*n* = 19

*Finger, handgrip, and pullup strength measured as extra weight/force in relation to body weight

**Subjects scored according to how far they were able to climb (best performance) before falling or successfully completing each route; § subjects scored according to best performance in five different problems

^§§^Subjects scored according to best performance out of three attempts

**Table 3:** Correlations between muscle strength, anthropometry, and climbing performance

	Self-reported best performance	Redpoint, score			Bouldering, total score	Campus board, score
		Route 1	Route 2	Total (1 and 2)		
Finger strength, kg	***r* = 0.76** *p* < 0.01	***r* = 0.65** *p* < 0.01	***r* = 0.59** *p* < 0.01	***r* = 0.67** *p* < 0.01	***r* = 0.89** *p* < 0.01	***r* = 0.82** *p* < 0.01
Handgrip strength, kg	*r* = 0.12 *p* = 0.62	*r* = 0.45 *p* = 0.05	***r* = 0.53** *p* < 0.05	***r* = 0.54** *p* < 0.05	*r* = 0.32 *p* = 0.19	***r* = 0.47** *p* < 0.05
Pullup strength, kg	*r* = 0.45 *p* = 0.05	*r* = 0.39 *p* = 0.10	*r* = 0.24 *p* = 0.32	*r* = 0.33 *p* = 0.17	***r* = 0.55** *p* < 0.05	***r* = 0.57** *p* < 0.05
Body weight, kg	*r* = − 0.29 *p* = 0.22	*r* = − 0.36 p = 0.14	*r* = − 0.45 *p* = 0.06	*r* = − 0.45 *p* = 0.06	***r* = **− **0.49** *p* < 0.05	***r* = **− **0.51** *p* < 0.05
Height, m	*r* = − 0.22 *p* = 0.36	*r* = − 0.07 *p* = 0.78	*r* = − 0.23 *p* = 0.34	*r* = − 0.18 *p* = 0.46	*r* = − 0.27 *p* = 0.28	*r* = − 0.25 *p* = 0.32
Body mass index, kg ∙ m^−2^	*r* = − 0.17 *p* = 0.50	*r* = − 0.44 *p* = 0.06	*r* = − 0.41 *p* = 0.08	***r* = **− **0.46** *p* < 0.05	*r* = − 0.42 *p* = 0.08	***r* = **− **0.48** *p* < 0.05

Pearson correlation coefficient used to determine association between muscle strength, anthropometrical, and climbing performance data. Correlations with a *p*-value < 0.05 highlighted in bold. *n* = 19

## Discussion

With the incremental growth in popularity of climbing there is a mounting demand for knowledge of physiological factors that are critical for performance and, in turn, which of these factors should be targeted in tailored training programs. The current study sought to investigate the role of forearm flexor muscle strength, perhaps intuitively the most limiting muscle group for climbing performance. The main findings were that 1) maximal isometric forearm flexor muscle strength exhibited very strong and strong associations with bouldering and redpoint, respectively. However, 2) the strength in these associations were present only if forearm muscle strength was assessed in a climbing-specific test, i.e., hanging from an edge with the distal finger joint. Isometric handgrip strength was associated only moderately with redpoint, but not bouldering performance. 3) Pullup strength showed moderate (bouldering) or no (redpoint) associations with climbing performance. 4) Body weight and BMI were moderately associated with bouldering and redpoint performance, respectively. The strong to very strong associations with redpoint and bouldering, explaining up to 80% of the performance variance in the current study, indicate that climbing-specific finger strength may be the most important physiological factor explaining steep climbing performance, at least when climbers have some time to practice the moves.

### Climbing-specific finger strength, forearm flexors, and climbing performance

We hypothesized that climbing-specific finger strength would strongly associate with both bouldering and redpoint climbing performance in moderate to high level climbers, while handgrip strength would not. As such, our findings are in line with our assumptions. The association between finger strength and climbing performance in the current study shows the importance of testing the forearm flexors in a climbing-specific grip position. Whereas climbing non-specific handgrip strength showed inconsistent, or at best moderate, association with performance (bouldering: n.s.; redpoint: ~ 30%); the proportion of variance in bouldering and redpoint that may be explained by climbing-specific finger strength in the present study is ~ 80% and 45%, respectively. A remarkably strong relationship, particularly for the former. Our result is, although with somewhat stronger correlation, in accordance with previous studies documenting relationships (*r* = 0.39–0.87) between hanging from 20 to 25 mm edges for ≤ 3 s and redpoint or/and bouldering performance in both males and females (Baláš et al. 2012; Giles et al. 2021). Several studies have also, without reporting associations, documented differences in climbing-specific finger strength between climbers and non-climbers (Fryer et al. 2015a, b; Grant et al. 2001; Vigouroux and Quaine 2006) as well as low- and high-level climbers (Fryer et al. 2015a, b; Ozimek et al. 2017). Importantly, bouldering and redpoint performances were predominantly performed at steep inclinations, and the climbers had 1.5 and 1 h to practice moves. This may have led performance to be less influenced of non-physiological factors, such as tactical and technical aspects, providing a stronger relationship to finger strength. Indeed, the very important role of climbing-specific finger strength was also observed during campus boarding performance, literally bouldering without using the lower limbs. Finally, the relatively similar results from self-reported performance and performance measured in the current study, also strengthens the confidence with which we can assume that climbing-specific finger strength may be the most critical physical component for steep climbing performance.

### Bouldering, redpoint, and climbing-specific finger strength

Interestingly, despite having a very different reliance on both anaerobic and aerobic endurance, bouldering and redpoint performance showed a close relationship (Fig. 3), indicating that they may both share key limiting factors. A likely important component is muscle strength, specifically climbing-specific finger strength, since endurance is minimally involved in bouldering. Indeed, the results from World Cups and World Championships the last decade have shown male and female climbers, e.g., Janja Garnbret and Adam Ondra, can win both categories, even in the same championship. Yet, climbing-specific finger strength is somewhat more strongly associated with bouldering than redpoint in the current study. This may not be particularly surprising, as previous studies have revealed a greater MVC (Fryer et al. 2017) and rate of force development (RFD) (Fanchini et al. 2013) in boulderers than lead climbers. In particular RFD is shown to be greater in boulderers, suggesting neural factors to be of key importance for bouldering and critical to meet the requirement for fast, powerful, movements of the discipline.

### Large muscle groups, pullup, and body weight

We also hypothesized that in contrast with climbing-specific finger strength, pullup strength, involving large muscle groups when pulling with the upper limbs, would not strongly associate with climbing performance. This is in line with our findings, i.e., the association between pullup strength and bouldering was only moderate and not present for redpoint, in the current study. The latter finding is in contrast some previous observations that elite climbers had superior pullup strength than recreational climbers (Grant et al. 1996), but in line with others showing no differences (Grant et al. 2001). The inconsistent, and at best moderate, relationship between pullup strength and climbing performance may be explained by the fact that muscle groups more proximal compared to the fingers become less important beyond a certain level of strength, i.e., when a climber has sufficient strength to pull to the next hold. It could also be explained by the size of the muscle groups involved. For the forearm flexors, hypertrophy does not represent a relevant cost in terms of increased body weight. However, for the larger back muscles, increasing hypertrophy and thus body weight may counteract the advantage of being stronger, especially since climbing-specific finger strength (i.e., extra weight in relation to body weight) will directly be influenced. In fact, body weight was negatively related to bouldering performance, and tended to be related to redpoint performance, in the current study. Such a relationship has also been reported previously (Ginszt et al. 2023).

### Practical implications and experimental considerations

Given the remarkably strong association between climbing-specific finger strength and bouldering, and to considerable extent redpoint too, appropriate testing should be a very useful tool for evaluating not only climbing ability, but also the effect of various training interventions. Notably, it is a very simple test that does not require advanced/expensive equipment or expertise and can be carried out also by the individual climbers themselves. Importantly, from a performance perspective, the strength test results should be given as additional kg over body weight in absolute values. This is because when the question is performance, different dimensions will inherently be a part of the climbers’ ability. It is the additional absolute weight over body weight that will determine the ability to hang onto an edge or, in accordance with Newton’s 2. law, to accelerate towards the next hold. Although it was not the scope of this investigation it may, however, be physiologically interesting to present values in relation to body weight to determine how impressive they are in some cases; for e.g., it is certainly more physiologically impressive for the larger individual to lift one’s body weight in a pullup or hang from an edge. It is possible that further details of various grip positions, i.e., crimp, half crimp, open hand, and pinch would provide additional information. The current study did not separate between these grips and thus represents a limitation of the results. Albeit, since the climbers could freely choose their grip of choice, it is likely that they chose their strongest grip suited for an edge and that this would be similar to what they would choose when climbing.

The present study was designed to document the relationship between muscle strength and climbing performance. It is thus limited to describing how much of the variance in bouldering/redpoint performance can be explained by climbing-specific finger, handgrip, and pullup strength. Future studies should investigate if, for e.g., climbing-specific finger strength training may improve climbing performance more than just simply climbing. With the recent development of the sport, perhaps in particular competition bouldering with parkour-like style and dynamic paddle moves, it may also be necessary to more accurately determine the role of climbing-specific finger strength in the disciplines that are more technically complex compared to the present study. Notably, however, self-reported outdoor climbing ability returned very similar results as in the two distinctly different climbing disciplines measured in the current study. Taken together, the results point towards climbing-specific finger strength as a critical component of overall climbing performance, and in some disciplines likely the most important one.

## Conclusions

The current study provides evidence that forearm strength may indeed be the physiological factor most strongly related to climbing ability, explaining up to 80% of the variation in performance, at least when climbers are given time to practice boulders and redpoint routes. However, of importance, forearm muscle strength should be tested in a climbing-specific test, e.g., when hanging from an edge with the distal finger joint. These findings emphasize the importance of appropriate testing and may ultimately influence how strength training is structured to target climbing-specific finger strength for performance enhancement in recreational and high-performing climbers.

## Data Availability

The datasets generated during and/or analyzed during the current study are available from the corresponding author on reasonable request.

## Abbreviations

1RM: One repetition maximum
BMI: Body mass index
IFSC: International Federation of Sport Climbing
n.s.: Not significant
Q-Q-plots: Quantile–Quantile plots
RFD: Rate of force development
SPSS: Statistical Package for the Social Sciences
